# Hydroxyketone Tyrosinase Inhibitors: Mechanism of Action, Applications and Perspectives in Depigmentation and Melanoma Therapy

**DOI:** 10.3390/molecules30204079

**Published:** 2025-10-14

**Authors:** Barbara Bogdańska, Dmytro Khylyuk, Dariusz Matosiuk

**Affiliations:** 1 Doctoral School, Medical University of Lublin, 20-093 Lublin, Poland; barbaradbogdanska@gmail.com; 2 Department of Synthesis and Chemical Technology of Pharmaceutical Substances, Faculty of Pharmacy, Medical University of Lublin, Chodzki 4A, 20-093 Lublin, Poland; 3 Department of Organic Chemistry, Faculty of Pharmacy, Medical University of Lublin, Chodzki 4A, 20-093 Lublin, Poland; dmytro.khylyuk@umlub.edu.pl

**Keywords:** tyrosinase inhibitors, molecular modeling, hydroxyketones, depigmentation, melanoma

## Abstract

Tyrosinase is a key enzyme in melanogenesis, playing an important role in skin, hair, and eye pigmentation, as well as in the enzymatic browning of fruits and vegetables. Excessive tyrosinase activity leads to hyperpigmentation and other dermatological problems, and it also causes losses in the food industry. For this reason, tyrosinase inhibitors have become the subject of intensive research in medicine, cosmetology, and food technology. Among various inhibitors, compounds containing ketone and hydroxyl groups draw special attention, because they have the ability to chelate copper ions in enzyme’s active center or block access to it. This article discusses the possible mechanisms of action, based on molecular modeling of interaction of PDB database retrieved model of enzyme with known natural inhibitors–kojic acid and tropolone, as well as 6-hydroxyimino derivatives of imidazo[1,2-a]imidazole-5-ones. The results suggest that the model of enzyme–ligand interaction can be useful in establishing affinity to tyrosinase of new natural and synthetic inhibitors, which can have broad applications in various fields, particularly in medicine and cosmetology, with promising prospects for further development.

## 1. Introduction

Tyrosinase, the key enzyme in melanin biosynthesis, is crucial for human skin, hair, and eye pigmentation. Its excessive activity contributes to dermatological disorders such as hyperpigmentation and to serious pathologies including melanoma. Moreover, tyrosinase is responsible for the enzymatic browning of fruits and vegetables. Consequently, the development of effective tyrosinase inhibitors has attracted considerable scientific interest in medicine, cosmetology, and food technology [1].

Among the many compounds studied for tyrosinase inhibition, those containing ketone and hydroxyl groups draw special attention. These functional groups enable effective chelation of copper ions present in the active center of the enzyme or access to catalytic site, which in both cases lead to the inhibition of its activity. Examples of such compounds are kojic acid, tropolone or α-hydroxyketones, which both exhibit competitive and mixed mechanisms of inhibition depending on the type of substrate and reaction conditions [2]. Many natural and synthetic hydroxy-ketones exhibit affinity toward tyrosinase and can be applied in reducing cosmetic or medical skin problems [3,4]. Recently, natural secondary metabolites such as flavonoids have been extensively investigated [5,6], while small molecules of synthetic origin have also been developed [7]. In addition to their anti-hyperpigmentation effects [8,9], numerous studies have also demonstrated anti-melanoma activity [10,11] and other cancer-related protective properties [12], particularly those associated with copper-containing enzymes. It is noteworthy that molecular modeling was frequently employed in these investigations [13,14].

**Figure 1:**
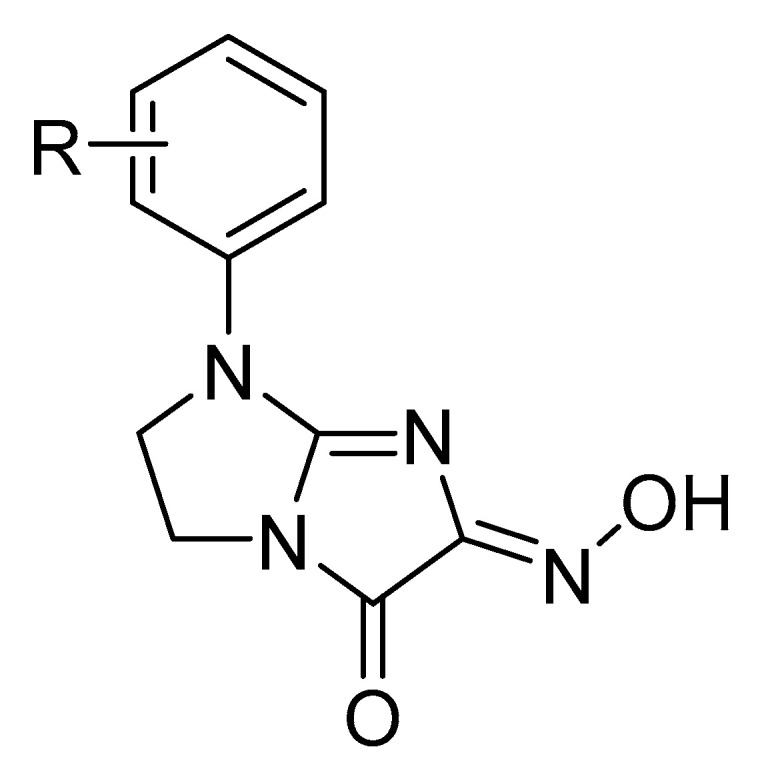
Structure of tested 1-aryl-6-iminohydroxy-5(1H)oxo-2,3-dihydroimidazo-[1,2-a]imidazoles. R=H, Cl (2-,3- or 4-), F (2-, 3- or 4-), CH_3_ (2-, 3- or 4-), CH_3_O (2-, 3- or 4-) (See Supplementary Material File S1).

This article discusses the mechanisms of action of tyrosinase inhibitors containing ketone and hydroxyl groups (Figure 1), with particular emphasis on their mode of interaction within the catalytic site, based on the results of molecular modeling. Additional attention has been devoted to their potential roles as depigmenting agents in cosmetology, antioxidants in food technology, and therapeutic compounds for treating disorders associated with hyperpigmentation. This article also discusses the prospects for developing new inhibitors based on these chemical scaffolds, as well as the challenges related to their application [15].

The use of computer-assisted methods in the design and development of biologically active compounds significantly accelerates the process and reduces associated costs [16,17]. Although favorable outcomes from modeling ligand–protein target interactions are crucial in drug discovery—particularly when artificial intelligence supports the process at this stage [17]—certain limitations still remain [18]. These include the necessity of validating modeling results with biological data or, at the very least, employing a training set of ligands with known activity toward a specific target [19].

Validation of experimental results represents a much broader challenge [20]. It can be carried out at either the mathematical [21,22] or biological [23] level. In our study, the mathematical approach was adopted through comparison with well-characterized natural ligands—kojic acid and tropolone. Another limitation concerns the lack of prediction of potential interactions with other biological targets, particularly those belonging to the same protein families. Such interactions can only be verified through extensive functional or behavioral studies using cell cultures or animal models.

Both depigmentation—requiring specifically targeted and safe cosmetic agents—and the hyperpigmentation observed in melanoma remain key objectives in ongoing research on tyrosinase inhibitors.

The epidemiology of melanoma shows significant geographical differences, with the highest incidence in Australia and New Zealand, as well as in the Scandinavian countries and North America. In recent decades, the incidence of melanoma has been steadily increasing, largely due to lifestyle changes that result in greater exposure to ultraviolet (UV) radiation. The primary risk factor for melanoma development is UV radiation, originating from both natural (sunlight) and artificial (tanning beds) sources. Episodes of intense tanning, especially in childhood and adolescence, are particularly dangerous [24].

For this reason alone, research on novel tyrosinase inhibitors is gaining increasing importance. Current studies focus on designing compounds with enhanced efficacy while maintaining low toxicity. The application of advanced computational chemistry techniques enables precise modeling of interactions between ketone and hydroxyl groups and the active site of tyrosinase, facilitating the development of highly selective inhibitors. In recent years, there has been growing interest in identifying tyrosinase inhibitors of natural origin. Plants, fungi, and microorganisms represent a rich source of potential inhibitors, and a detailed understanding of their interaction mechanisms can provide valuable insights for the design of new synthetic compounds with improved potency and specificity [25].

Tyrosinase inhibitors act as functional antagonists of the tyrosinase enzyme, which regulates the rate of melanin synthesis, leading to reduced melanin production in melanoma cells and potentially limiting tumor growth [26]. Both in vitro and in vivo studies have demonstrated that tyrosinase inhibitors not only suppress melanin biosynthesis but also enhance the sensitivity of melanoma cells to chemotherapy and radiotherapy, thereby improving therapeutic efficacy. Compounds such as kojic acid, arbutin, and tropolone are currently being investigated as potential anticancer agents for melanoma treatment [27]. Moreover, the combination of tyrosinase inhibitors with other therapeutic strategies—such as BRAF and MEK inhibitors—is under active investigation [28].

### The Role of Ketone and Hydroxyl Groups

The ketone group (C=O) is particularly effective in chelating copper ions present in the active site of tyrosinase as well as being included in the hydrogen bonds network with amino acids within the catalytic site. This disruption of the active site structure prevents the enzyme from catalyzing oxidative reactions, such as the conversion of L-tyrosine to L-DOPA or L-DOPA to dopaquinone [29,30]. An example of a compound utilizing a keto group to inhibit tyrosinase is kojic acid (KA). Kojic acid exhibits a mixed mechanism of tyrosinase inhibition, as it can bind both to the enzyme’s active site and to additional allosteric sites. This dual interaction results in a reduction in the maximum reaction velocity (Vmax) and an increase in the Michaelis constant (Km) [29].

Hydroxyl groups (-OH) also play an important role in tyrosinase inhibition. They can form hydrogen bonds with amino acids in the active site of tyrosinase or interact directly with copper ions [31,32]. The number and position of hydroxyl groups in aromatic rings significantly affect the activity of the inhibitor. Studies have shown that compounds with multiple hydroxyl groups, such as butein, exhibit stronger tyrosinase inhibition than compounds with a single hydroxyl group [32]. This observation suggests that the presence of multiple hydroxyl groups may enhance the ability of the inhibitor to form stable complexes with the enzyme’s active site [33].

## 2. Results

The validation procedure confirmed the ability of the selected software and used parameters to reproduce the position of the ligands inside the binding site with a sufficient level of accuracy (RMSD = 1.252).

**Figure 2:**
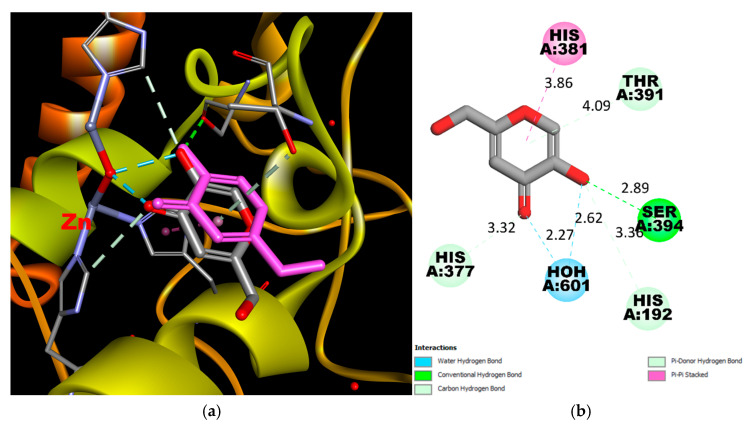
(**a**) Real and Predicted Positions of Kojic Acid in the Binding Site. A comparison between the experimentally determined position and the computationally predicted position of kojic acid within the active site of Tyrosinase-Related Protein 1 (TYRP1). The real position is based on crystallographic data, while the predicted position was obtained through molecular docking simulations. (**b**) 2D Interaction Scheme of Kojic Acid in the Binding Site. A schematic representation of the interactions between kojic acid and key residues in the TYRP1 binding site. This diagram was generated using the PDB structure 5M8M and visualized with Discovery Studio Visualizer. Interactions include hydrogen bonding between the hydroxyl and keto groups of kojic acid and the residues in the active site, without direct coordination with the zinc ions.

**Figure 3:**
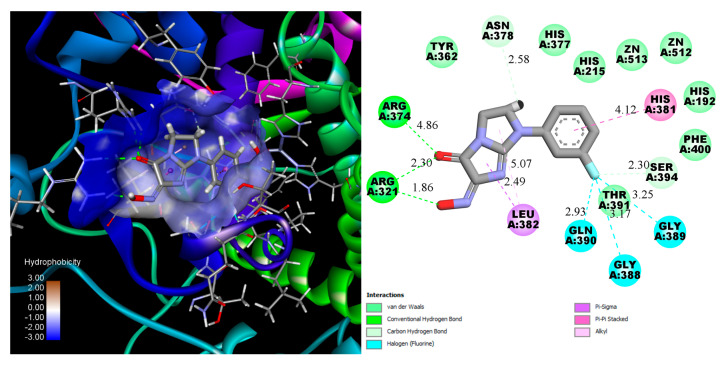
3D and 2D schemes of the interaction of compound **12** with tyrosinase (PDB ID: 5M8M).

Docking studies revealed the high potential of the entire compound series, as evidenced by higher docking scores compared to the reference ligands (Figure 2). The best performance was observed for compound **12,** which contains a 3-fluorophenyl substituent (Figure 3).

Across compounds **1**–**13**, which share a common 6-hydroxyimino-imidazo [1,2-a]imidazol-5-one core and differ only by the aryl substituent on the imidazoline ring, the docking scores demonstrate a clear dependence on both substituent type and position. Halogenated derivatives exhibited the best overall performance, with the meta-fluoro analogue 12 showing the most favorable score (≈−23.5), followed by para-fluoro 11 (≈−21.7). Among electron-donating substituents, the meta-methoxy derivative 6 was also highly ranked (≈−21.9), indicating that strong binding performance is not restricted to halogen substituents but is enhanced by groups that balance polarity and polarizability with compact steric profiles. In contrast, ortho-substitution generally reduced activity, consistent with steric hindrance near the aryl–imidazoline linkage; the ortho-methyl 2 was the weakest compound in the series (≈−11.5). The unsubstituted phenyl 1 displayed intermediate behavior (≈−18.4). Taken together, the observed trend is meta ≥ para » ortho, with F (and, secondarily, Cl) > OMe ≳ Me > H. Relative to the reference inhibitors, most analogues (except 2) outperformed kojic acid (≈−16.5) and tropolone (≈−11.7), supporting the conclusion that appropriately substituted aryl groups—particularly meta-F—optimize the predicted binding affinity within this chemotype (Table 1).

Compound **12** forms hydrogen bond with Arg374, measuring 4.86 Å, and two strong hydrogen bonds with Arg321 with the short distances near 2.30 Å and 1.86 Å. The 3-fluoro substituent anchors in the cavity by three halogen non covalent interactions with Gln390, gly388 and Gly389. Additionally, Leu382 and His381 interact with the imidazo [1,2-a]imidazol-5-one and phenyl cores, further enhancing the binding stability of the ligand. A number of amino acids interact with the ligand by weak van der Waals force.

It should be noted that there are no direct donor-acceptor interactions between the Zn ion and compounds. However, kojic acid and tropolone also bind near the metal ions, approximately 3 Å away, which is too distant for effective coordination [30]. Despite the lack of direct coordination, their proximity to zinc ions still allows them to interfere with the enzyme’s catalytic activity, contributing to their inhibitory effects. Therefore, the high docking scores, which exceed those of the reference ligands, suggest that the synthesized compounds may bind strongly to tyrosinase and compete with its natural substrates, such as tyrosine or DOPA, for access to the enzyme’s active site.

Considering that existing and potential tyrosinase inhibitors must physically hinder substrate entry into the enzyme’s active site, the stability of enzyme-inhibitor complexes is a key factor. Molecular dynamics simulations were performed using the GROMACS software package for a simulation time of 100 ns, which is sufficient to evaluate the stability of the complexes. Additionally, molecular dynamics simulations were conducted using kojic acid as a reference molecule. The stability of both complexes was compared using standard parameters, including root mean square deviation (RMSD), root mean square fluctuation (RMSF), the number of hydrogen bonds, and the radius of gyration (Rg).

**Figure 4:**
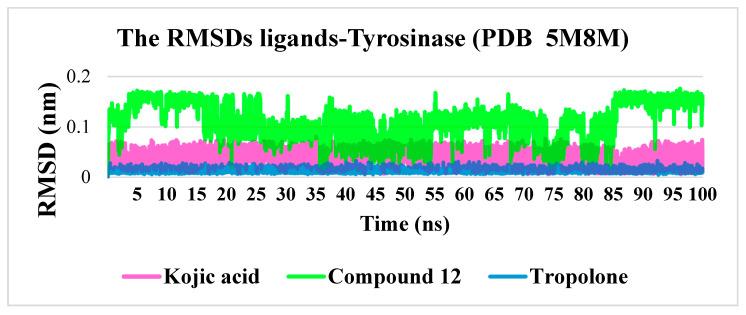
Protein–ligand RMSDs for kojic acid, Tropolone and compound **12**.

The RMSD values demonstrated a sufficient level of stability for the tyrosinase–compound **12** complex, as the RMSD did not exceed 0.3 nm. At its peak, the RMSD reached an average value of approximately 0.16 nm between 4–15 ns and 85–100 ns. Notably, the reference compounds, kojic acid and tropolone, exhibited exceptional complex stability, with RMSD values of approximately 0.05 nm and 0.015 nm, respectively. This remarkable stability is attributed to the planar structure of kojic acid and tropolone molecules, which have either a single torsion point or none (in the case of tropolone), thereby reducing molecular fluctuations after binding within the active site (Figure 4).

RMSF is a metric used to assess the average amplitude of atomic or residue-level fluctuations in a molecule during a molecular dynamics simulation. Typically, the C- and N-termini of proteins exhibit greater fluctuations compared to the core regions due to their higher flexibility. In contrast, secondary structure elements such as α-helices and β-sheets are generally more rigid and show lower fluctuations compared to disordered or unstructured regions of the protein.

**Figure 5:**
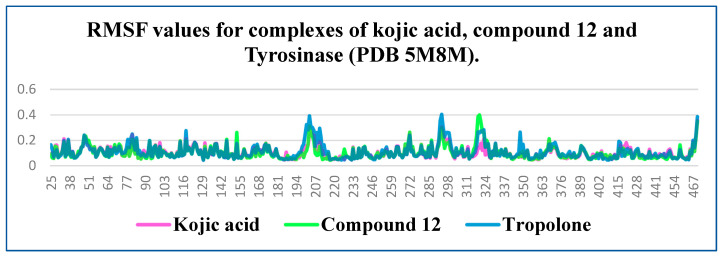
The calculated average RMSF for tyrosinase with kojic acid, tropolone and compound **12**.

In protein–ligand systems, low RMSF values in the binding region often indicate complex stability, as reduced fluctuations suggest that the ligand is firmly anchored within the active site. This relationship underscores the importance of RMSF analysis in understanding molecular flexibility and the dynamics of biomolecular interactions. RMSF plots reveal minor differences among all three complexes, particularly in the binding region (residues 360–391). This observation suggests comparable stability among the complexes (Figure 5).

The protein RMSF of Tyrosinase in complex with the kojic acid (pink colored), Tropolone (blue colored) and compound **12** (green colored).

Hydrogen bonds play a crucial role in stabilizing protein structures and ligand-protein complexes. During molecular dynamics simulations, analyzing the number and stability of these bonds helps assess the strength of ligand-protein interactions, which serves as a key indicator of binding stability.

Hydrogen bonds between the ligand and protein help anchor the ligand in the active site or another specific protein region. A stable number of hydrogen bonds throughout the simulation indicates reliable binding.

**Figure 6:**
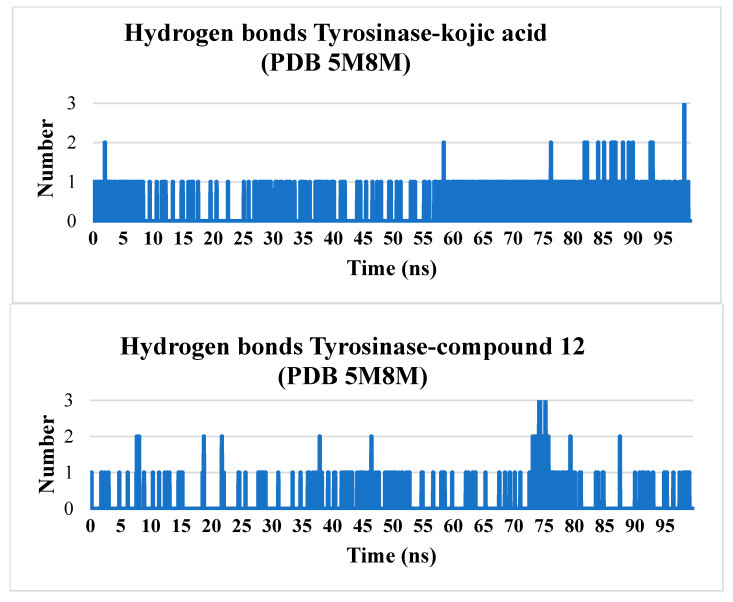

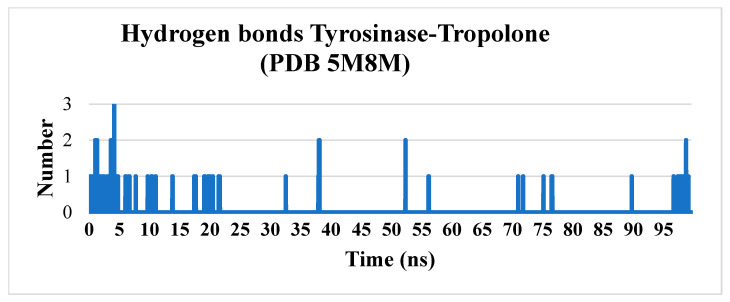
The number of hydrogen bonds during the simulation for complexes of tyrosinase with kojic acid, tropolone and compound **12**.

Kojic acid and compound **12** form hydrogen bonds with the protein, but kojic acid maintains a more stable number of hydrogen bonds throughout the simulation. Compound **12** forms, on average, one to two hydrogen bonds, with intermittent periods where no hydrogen bonds are observed. Tropolone, owing to its simple single-phenyl core scaffold, typically does not form hydrogen bonds but occasionally forms one to three hydrogen bonds (Figure 6).

**Figure 7:**
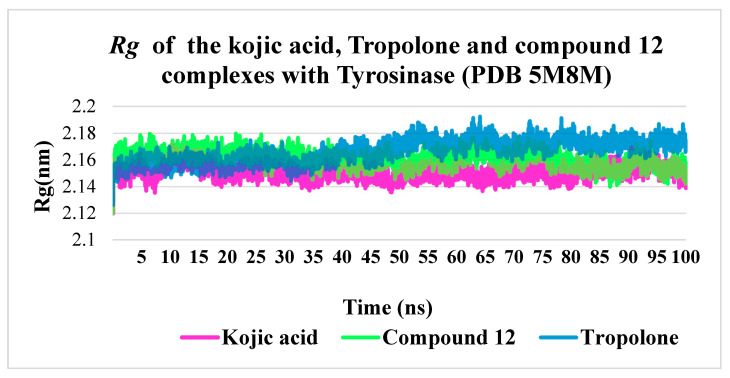
The ***Rg*** values for kojic acid, tropolone and compound **12** complexes with tyrosinase.

The radius of gyration (***Rg***) quantitatively measures the compactness or spatial distribution of a molecule’s atoms relative to its center of mass. Consequently, the trajectory analysis of ***Rg*** reflects changes in the overall dimensions of the protein throughout the molecular dynamics simulation. The average ***Rg*** value for the kojic acid–tyrosinase complex was 2.15 nm, while the complex with compound **12** exhibited a slightly higher value (2.16–2.17 nm). Similarly, the tropolone–tyrosinase complex demonstrated comparable stability, with slightly elevated ***Rg*** values from 50 ns onward. Ultimately, all three complexes remained stable over the 100 ns simulation period, indicating comparable compactness and stability under the given simulation conditions (Figure 7).

The MM-PBSA results (Table 2) indicate that compound **12** exhibits the most negative binding free energy, signifying its strong affinity for the tyrosinase active site. Van der Waals interactions contribute substantially to this enhanced binding, with electrostatic components also playing a key role. In contrast, tropolone shows a less negative free energy despite its planar structure and low RMSD values, suggesting fewer stabilizing hydrogen bonds. Kojic acid demonstrates moderate binding energy, reflecting a balance between electrostatic and solvation effects. Overall, these findings highlight the binding potential of compound **12**, warranting further investigation as a tyrosinase inhibitor.

### HOMO–LUMO Transition and Biological Activity

**Figure 8:**
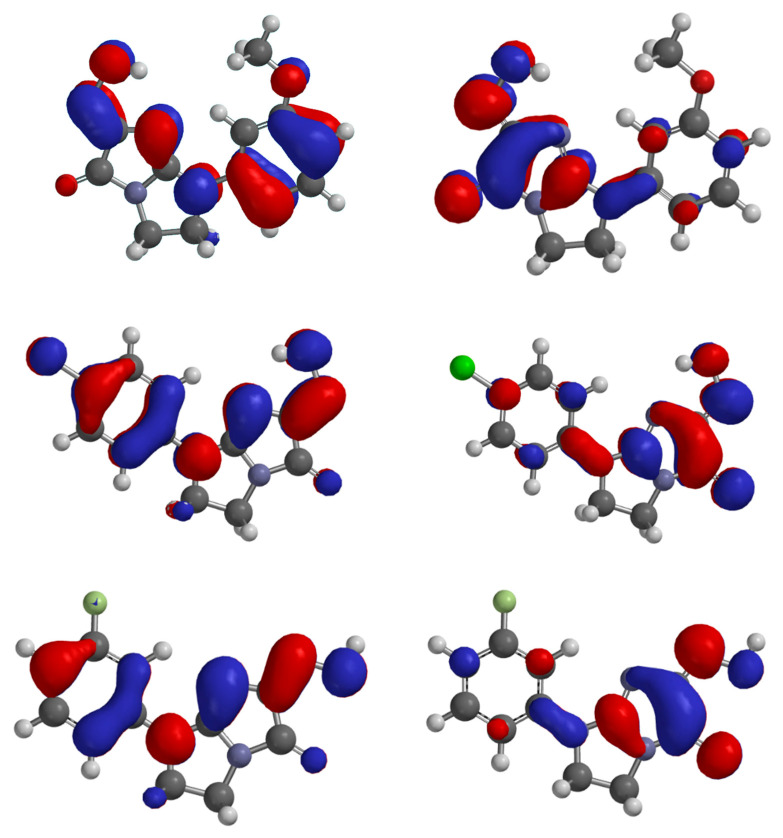

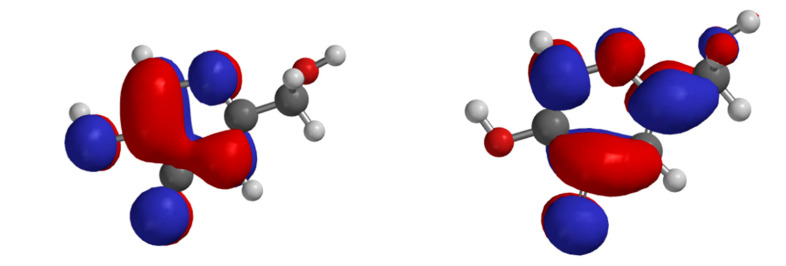
HOMO, LUMO of the compound **6**, **8**, **12** and kojic acid.

The calculated HOMO–LUMO energy gap, together with the derived reactivity descriptors, provides insight into the potential bioactivity of the proposed compounds. HOMO level reflects the electron-donating capacity of the molecule, which is critical for coordinating with the metal center, while LUMO indicates the ability to accept electrons from the enzymatic environment (Figure 8). A moderate HOMO–LUMO gap facilitates these interactions, potentially enhancing inhibitory potency.

Frontier-orbital and global-reactivity indices derived from our B3LYP calculations delineate clear electronic differences among compounds **1**–**13** (Table 3).

The HOMO–LUMO gaps of the studied compounds (0.147–0.184 a.u.) indicate that all molecules possess sufficient electronic softness to engage in charge transfer with the copper ions in the tyrosinase active site. Compound **2** has the narrowest gap and thus the highest electronic flexibility, making it a strong potential electron donor. In contrast, compound **9** has the widest gap, making it the hardest and least likely to adapt to the enzyme’s electronic environment.

The ionization potential (related to the molecule’s ability to donate electrons) is lowest for compounds **2** and **5**, suggesting they are well-suited to transfer electron density toward the metal center. All analogues have higher electron affinities than kojic acid, which means they are more capable of accepting electrons from the enzymatic surroundings—an important feature for forming a stable complex with Cu(I).

Compounds **8** (ω = 0.134 a.u.) and **6** (ω = 0.133 a.u.) exhibit the most favorable electronic profiles—high electrophilicity and suitable softness—facilitating charge transfer and coordination with the copper center essential for tyrosinase inhibition. In contrast, kojic acid shows lower electrophilicity (ω = 0.120 a.u.), while compounds **7** and **9** appear less promising due to low electrophilicity and excessive hardness, respectively.

Collectively, the descriptor pattern yields a predicted reactivity hierarchy of **8** ≈ **6** > **2** > **1** ≈ **3** ≈ **10** ≈ **11** ≈ **12** > **5** > **13** > **4** > **9** > **7**.

## 3. Materials and Methods

With the aim to explore and compare the potential interactions of the synthesized compounds with the tyrosinase enzyme molecular docking studies were conducted. The three-dimensional structure of the target enzyme was obtained from the Protein Data Bank (PDB ID: 5M8M) [34]. FlexX docking program from the LeadIT 2.3.2 software package was chosen for the in silico simulations due to its accuracy in predicting the native ligand’s binding position within the active site, with acceptable RMSD values. Additionally, FlexX’s capability to predict interactions with metal ions, which may be crucial for inhibition activity, was a key factor in its selection [35]. The radius of the binding site was expanded from the standard 6.5 Å to 9 Å. The zinc ion located within the binding site was included in the simulation region. The structures of the investigated compounds were minimized using Avogadro v. 1.2.0. software, applying molecular mechanics with the MMFF94 force field over 10,000 cycles [36]. The native ligand, kojic acid, was utilized for the redocking validation procedure to confirm the reliability of the selected docking parameters [37]. Additionally, tropolone, a known experimentally validated tyrosinase inhibitor, was included in the compound set [38]. The docking scores obtained for kojic acid and tropolone were subsequently used as reference points for quantitative comparison with the synthesized compounds. Biovia Discovery studio visualizer v21.1 was used for the visualization of the obtained results.

The GROMACS 2021 software package implemented into the SibioLead server were used to perform molecular dynamics simulations [39]. The complexes were carefully placed within a cubic simulation box and solvated using the SPC water model to mimic aqueous conditions. The force field AMBERSS9B was chosen for performing the simulations [40]. The Na^+^ and Cl^−^ ions were added to the system at a concentration of 0.15 M for neutrali zation. Energy minimization was performed using the steepest descent algorithm, consisting of 5000 steps to optimize the system’s potential energy landscape. Following minimization, the system was equilibrated under canonical (NVT) and isothermal–isobaric (NPT) ensembles at a constant temperature of 300 K and pressure of 1 bar for 1000 ps. This equilibration phase prepared the system for the production run. The production phase of molecular dynamics was carried out over 100 ns using the Leapfrog integrator, with trajectory data collected as 5000 discrete frames for detailed analysis.

The binding free energy (ΔG binding) of protein–ligand complexes was calculated at 2-nanosecond intervals throughout the entire 100 ns simulation, resulting in 50 extracted frames from the GROMACS simulation. The estimates were obtained using an automated plugin for the Molecular Mechanics Poisson-Boltzmann Surface Area (MM-PBSA) method via the SiBioLead server. The results were subsequently analyzed and visualized using Microsoft Excel.

The frontier molecular orbitals, namely the highest occupied molecular orbital (HOMO) and the lowest unoccupied molecular orbital (LUMO), were calculated using Spartan 24. The optimized structures obtained before were used for the calculations. Subsequently, geometry optimizations were performed using Density Functional Theory (DFT) with the B3LYP hybrid functional and the 6-31G(d,p) basis set on the gas-phase optimized geometries [41].

Following optimization, a single-point energy calculation was conducted at the same level of theory to obtain the molecular orbital energies. All orbital energies were retained in atomic units (a.u.; 1 a.u. = 1 Hartree = 27.2114 eV). The same unit system was used to derive and report the global reactivity descriptors—vertical ionization potential (*I* = −E_HOMO_), electron affinity (*A* = −E_LUMO_) [42], chemical hardness (η = ΔE/2) [43], global electronegativity (χ = (*I* + *A*)/2) [44] and electrophilicity index (ω = χ^2^/2η) [45] ensuring internal consistency and avoiding unit-conversion artifacts.

## 4. Conclusions

Comprehensive in silico profiling has revealed the d library to be a fertile source of new tyrosinase inhibitors. Flexible docking identified ten of the thirteen synthesized molecules as energetically superior to the reference ligands, with compound **12** achieving the most favorable FlexX score −23.5156 and exhibiting an extensive hydrogen-bond/halogen-bond network within the 5M8M binding cleft. One-hundred-nanosecond molecular-dynamics simulations corroborated the reliability of the docking poses: the tyrosinase–12 complex remained structurally stable (RMSD ≤ 0.16 nm) and retained a compact global fold (***Rg*** ≈ 2.16 nm) comparable to that of the kojic-acid complex. MM-PBSA analysis further ranked 12 as the strongest binder (ΔG= −15.8 ± 3.1 kcal mol^−1^), with van der Waals and electrostatic components jointly dominating the interaction energy.

Frontier-orbital calculations at the B3LYP/6-31G(d,p) level showed HOMO–LUMO gaps of 0.147–0.184 a.u. for all analogues, confirming sufficient electronic softness for π → π* or *n* → π* charge transfer. Global reactivity descriptors singled out compounds **8** and 6 (ω ≈ 0.134 a.u.) as the most electrophilic, suggesting a strong propensity for Cu-centered redox interactions. Although 12 is electronically mid-ranking (ω = 0.126 a.u.), its superior shape complementarity and multivalent non-covalent contacts appear to offset this, yielding the most stable enzyme complex in silico.

Taken together, the data delineate two complementary optimization avenues: (i) compound **12** as a lead whose favorable binding geometry can be fine-tuned to reinforce hydrogen-bond persistence, and (ii) compounds **8** and **6** as electronically privileged scaffolds that merit structural elaboration aimed at enhancing steric fit. The convergence of docking, MD stability, free-energy estimations and quantum-chemical descriptors underscore the overall promise of the series and provide a rational basis for prioritizing synthetic expansion and biochemical validation.

In summary, tyrosinase inhibitors, especially those containing ketone and hydroxyl groups, show significant potential in various fields. In cosmetology, their ability to reduce hyperpigmentation makes them valuable ingredients in skin care products. In the food industry, they can prevent undesirable enzymatic browning, improving the quality and shelf life of products. Most importantly, tyrosinase inhibitors show promising prospects in the treatment of melanoma, an aggressive skin cancer.

Melanoma is a major health problem worldwide, and its early detection and effective treatment are crucial to improve patient prognosis. Therefore, research on tyrosinase inhibitors that can inhibit melanoma cell growth and proliferation is extremely important. Compounds such as kojic acid, arbutin, and tropolone, due to their chelating and inhibitory properties, are promising candidates for anticancer drugs.

However, the development of effective melanoma therapies based on tyrosinase inhibitors requires further intensive research. To confirm the accuracy of the modeling results validation studies on cell lines are necessary. Such investigations are planned in the near future, focusing on both enzyme inhibition and anti-melanoma activity in cell models. Preliminary results have confirmed the inhibitory effect on tyrosinase. The research will be continued, and the results will be published. Preclinical trials evaluating the efficacy and safety of these compounds are also planned.

Future research should focus on:

- Designing new tyrosinase inhibitors with better pharmacokinetic and pharmacodynamic properties.
- Studying the mechanisms of action of tyrosinase inhibitors at the molecular and cellular level.
- Evaluating the efficacy of combinations of tyrosinase inhibitors with other anticancer therapies, such as immunotherapy and targeted therapies.
- Identifying biomarkers that will predict patient response to tyrosinase inhibitors.

## Figures and Tables

**Table 1:** The docking score of the synthesized set and reference ligands.

Compound		FlexX Docking Score (Human Tyrosinase-Related Protein 1—PDB 5M8M)
**1**	H	−18.3830
**2**	2-CH_3_	−11.4455
**3**	3-CH_3_	−18.1416
**4**	4-CH_3_	−20.6370
**5**	2-CH_3_O	−19.2495
**6**	3-CH_3_O	−21.8964
**7**	4-CH_3_O	−21.2831
**8**	2-Cl	−18.2830
**9**	3-Cl	−18.6898
**10**	4-Cl	−19.0834
**11**	2-F	−21.6780
**12**	3-F	−23.5156
**13**	4-F	−20.9563
**Kojic acid**		−16.5135
**Tropolone**		−11.7053

**Table 2:** MM-PBSA binding-energy decomposition for tyrosinase (PDB ID 5M8M) in complex with kojic acid, compound **12** and tropolone.

Complex	ΔG	Van der Waal Energy	Electrostatic Energy	Polar Solvation Energy	Non-Polar Solvation Energy	Solvation Energy
5M8M- kojic acid	−13.41 ± 2.92	−23.37 ± 2.03	−5.41 ± 2.04	15.37 ± 3.31	−1.84 ± 0.04	15.36 ± 3.31
5M8M- Compound **12**	−15.80 ± 3.10	−25.70 ± 2.10	−7.00 ± 2.10	18.60 ± 3.40	2.00 ± 0.08	16.60 ± 3.40
5M8M- Tropolone	−11.50 ± 2.20	−20.20 ± 2.00	−3.40 ± 1.90	13.40 ± 2.50	−1.60 ± 0.26	11.80 ± 2.50

**Table 3:** Frontier-orbital energies and global reactivity descriptors for compounds **1**–**13** and kojic acid calculated at the B3LYP/6-31G(d,p) level; all values are given in atomic units (1 a.u. = 27.2114 eV).

Compound	E_HOMO (a.u.)	E_LUMO (a.u.)	ΔE (a.u.)	η (a.u.)	I (a.u.)	A (a.u.)	χ (a.u.)	ω (a.u.)
**1**	−0.22417	−0.06247	0.1617	0.08085	0.22417	0.06247	0.14332	0.12704
**2**	−0.2058	−0.0588	0.147	0.0735	0.2058	0.0588	0.1323	0.11907
**3**	−0.2205	−0.06247	0.15802	0.07901	0.2205	0.06247	0.14148	0.12668
**4**	−0.2205	−0.05512	0.16537	0.08269	0.2205	0.05512	0.13781	0.11484
**5**	−0.20947	−0.0588	0.15067	0.07534	0.20947	0.0588	0.13413	0.11941
**6**	−0.21682	−0.06615	0.15067	0.07534	0.21682	0.06615	0.14148	0.13286
**7**	−0.21682	−0.04777	0.16905	0.08452	0.21682	0.04777	0.1323	0.10354
**8**	−0.22785	−0.06615	0.1617	0.08085	0.22785	0.06615	0.147	0.13363
**9**	−0.22785	−0.06982	0.18375	0.09187	0.22785	0.06982	0.14883	0.12056
**10**	−0.22417	−0.06247	0.1617	0.08085	0.22417	0.06247	0.14332	0.12704
**11**	−0.21315	−0.06247	0.15067	0.07534	0.21315	0.06247	0.13781	0.12605
**12**	−0.21682	−0.06247	0.15435	0.07717	0.21682	0.06247	0.13965	0.12635
**13**	−0.2205	−0.0588	0.1617	0.08085	0.2205	0.0588	0.13965	0.1206
Kojic acid	−0.23152	−0.04042	0.15435	0.07717	0.23152	0.04042	0.13597	0.11979

*E_HOMO*—energy of the highest occupied molecular orbital; *E_LUMO*—energy of the lowest unoccupied molecular orbital; Δ*E* = *E_LUMO* − *E_HOMO* (band gap); *η* = Δ*E*/2 (chemical hardness, a measure of resistance to charge deformation); *I* = −*E_HOMO* (vertical ionization potential, approximating electron-donor ability); *A* = −*E_LUMO* (vertical electron affinity, reflecting electron-acceptor strength); *χ* = (*I* + *A*)/2 (global electronegativity, overall tendency to attract charge); *ω* = *χ*^2^/(2*η*) (electrophilicity index, the energetic stabilization achievable upon accepting electrons).

## Data Availability

Structural data can be found in the Supplementary Materials.

## Supplementary Materials

The following supporting information can be downloaded at: https://www.mdpi.com/article/10.3390/molecules30204079/s1. File S1: information on structure of the elaborated compound in form of the SMILE files.
