# Supplementary material for: Hydroxyketone Tyrosinase Inhibitors: Mechanism of Action, Applications and Perspectives in Depigmentation and Melanoma Therapy
Source: Molecules. 2025 Oct 14;30(20):4079. doi: 10.3390/molecules30204079 (PMC12566265; doi:10.3390/molecules30204079)
Supplement: Supplementary file 1 [file molecules-30-04079-s001.zip › molecules-3871552-supplementary File S1.pdf]

1. O\N=C/1\N=C2N(CCN2c3ccccc3)C1=O
2. Cc1ccc(cc1)N2CCN3C(=O)\C(=N/O)\N=C23
3. Cc1cccc(c1)N2CCN3C(=O)\C(=N/O)\N=C23
4. Cc1ccccc1N2CCN3C(=O)\C(=N/O)\N=C23
5. COc1ccc(cc1)N2CCN3C(=O)\C(=N/O)\N=C23
6. COc1cccc(c1)N2CCN3C(=O)\C(=N/O)\N=C23
7. COc1ccccc1N2CCN3C(=O)\C(=N/O)\N=C23
8. O\N=C/1\N=C2N(CCN2c3ccc(Cl)cc3)C1=O
9. O\N=C/1\N=C2N(CCN2c3cccc(Cl)c3)C1=O
10. O\N=C/1\N=C2N(CCN2c3ccccc3Cl)C1=O
11. O\N=C/1\N=C2N(CCN2c3ccc(F)cc3)C1=O
12. O\N=C/1\N=C2N(CCN2c3cccc(F)c3)C1=O
13. O\N=C/1\N=C2N(CCN2c3ccccc3F)C1=O
